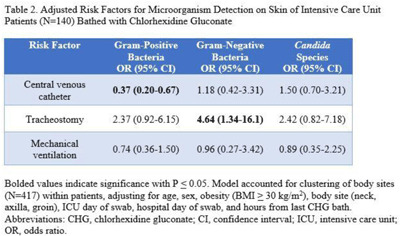# Indwelling medical devices and skin microorganisms on ICU patients bathed with chlorhexidine gluconate

**DOI:** 10.1017/ash.2022.139

**Published:** 2022-05-16

**Authors:** Yoona Rhee, Mary Hayden, Michael Schoeny, Christine Fukuda, Pamela B. Bell, Andrew Simms, Beverly Sha, Carlos Santos, Michael Lin

## Abstract

**Background:** Bathing ICU patients with chlorhexidine gluconate (CHG) decreases bloodstream infections and multidrug-resistant organism transmission. The efficacy of CHG bathing on skin microorganism reduction may be influenced by patient-level clinical factors. We assessed the impact of clinical factors on the recovery of microorganisms from the skin of patients admitted to an ICU who were receiving routine CHG bathing. **Methods:** We analyzed data obtained from 6 single-day point-prevalence surveys of adult ICU patients between January and October 2018 at 1 medical ICU, in the context of a CHG bathing quality initiative. Demographics and covariates were collected at the bedside and by chart review. Skin swabs were collected from neck, axilla, and inguinal regions and were plated to selective and nonselective media. Standard microbiologic methods were used for species identification and susceptibilities. Multivariable models included patients who received a CHG bath and accounted for clustering of body sites within patients. **Results:** Across all time points, 144 patients participated, yielding 429 skin swab samples. Mean age was 57 years (SD, 17); 49% were male; 44% had a central venous catheter; and 15% had a tracheostomy Also, 140 patients (97%) had >1 CHG bath prior to skin swab collection, with a median of 9 hours since their last CHG bath (IQR, 6–13 hours). Gram-positive bacteria were more commonly recovered than gram-negative or *Candida* spp across all skin sites (Table 1). Variation by body site was detected only for gram-positive bacteria, with recovery more common from the neck compared to axilla or groin sites. On multivariate logistic regression (Table 2), presence of central venous catheter was associated with lower odds of gram-positive bacteria recovery among those who received a CHG bath. Presence of tracheostomy was associated with a significantly higher odds of gram-negative bacteria detection on skin. No clinical factors were independently associated with recovery of *Candida* spp. **Conclusions:** Central venous catheter presence was associated with lower odds of gram-positive bacteria detection on skin, suggesting the possibility of higher quality CHG bathing among such patients. Tracheostomy presence was associated with greater odds of gram-negative bacteria detection, suggesting that it may be a potential reservoir for skin contamination or colonization. Indwelling medical devices may influence CHG bathing effectiveness in reducing microorganism burden on skin.

**Funding:** None

**Disclosures:** None

**Table:**
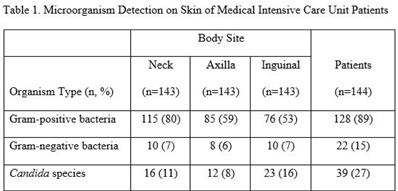

**Table:**